# One Welfare in Europe: Cross-country comparison of citizens’ and supply chain actors’ perceptions

**DOI:** 10.1017/awf.2026.10114

**Published:** 2026-09-28

**Authors:** Charlotta Harju, Katriina Heinola, Minna Väre, Jarkko Niemi

**Affiliations:** Natural Resources Institute Finland, Finland

**Keywords:** Animal welfare, citizen perceptions, food value chain, One Welfare, stakeholder perceptions, survey methodology

## Abstract

Animal welfare is closely linked to human health and the environment. Over the last decade, One Welfare (OW) has emerged to address these connections. However, the academic research on OW, which integrates human well-being, animal welfare, and the environment, is still in its early stages, with limited insights into how it is perceived by supply chain actors. This study aims to explore how European citizens and other actors perceive the OW construct, particularly in relation to improved farm animal welfare. The data were collected in 2021 in two surveys conducted in nine European countries (Finland, the UK, France, Italy, Belgium, Germany, the Netherlands, Romania, and Denmark) targeted at citizens (n = 3,601) and other supply chain actors (n = 222). Exploratory factor analysis (EFA) and a non-parametric Kruskal-Wallis test were employed to analyse the perceptions among the countries. The results show that supply chain actors have a more multidimensional understanding of OW and farm animal welfare than citizens. Citizens viewed OW as a two-dimensional construct related to human well-being, farming success and food security, while supply chain actors perceived it as three-dimensional, also including issues related to environmental sustainability. Statistically significant differences were found across certain countries. For example, Italian citizens associated OW primarily with human well-being, while Romanian citizens linked it to farming success. However, both Italian and Romanian supply chain actors perceived food safety and environmental sustainability as most associated with OW. Understanding these perceptions is essential for developing policies that support public health, sustainability, and ethical practices across Europe.

## Introduction

Animal welfare has clear connections with animal and human health and the environment (de Passillé & Rushen 2005; Goldberg 2016). Promoting animal welfare can reinforce sustainability goals such as food security, biodiversity conservation, productivity in agriculture, and human health (United Nations 2024). Furthermore, many health issues arise from poor animal and human welfare and environmental sustainability (Racciatti *et al.* 2023).

In both practice and research, concepts such as One Health (e.g. Jordan & Lem 2014; Radeski *et al.* 2018) and One Welfare (Colonius & Earley 2013; Jordan & Lem 2014; García Pinillos *et al.* 2016), have emerged to describe the interlinkages between animal and human health and the environment. One Welfare (OW) is a holistic perspective referring to the interconnection of human well-being, animal welfare, and the environment, and the concept seeks to enhance both animal welfare and human well-being at a global scale (García Pinillos *et al.* 2016). It also promotes the link between animal welfare and human welfare, extending and overlapping with the approach of One Health. The One Health (OH) is a concept that highlights the interconnected approaches to health, emphasising the commonalities of human, animal, plant, and environmental health (Radeski *et al.* 2018).

Animal welfare is a multidimensional concept (e.g. Lipovšek *et al.* 2024), including aspects of the physical health, emotional well-being, and appropriate behaviour of animals (Fraser *et al.* 2008). Industrialised high-density farming systems for any livestock species tend to share common traits that often lead to animal welfare issues, especially in relation to providing necessary resources and managing the animals in ways that meet their specific needs (de Passillé & Rushen 2005). For example, these characteristics are linked with housing design, feed and water, bedding, and other conditions, as well as to animal handling and treatments, such as pain management (e.g. Tarazona *et al.* 2019).

The European Union (EU) prioritises the enforcement of current standards, increasing awareness, and creating risk assessment criteria for farm animal welfare (Radeski *et al.* 2018). There are voluntary farm animal welfare schemes in many European countries (e.g. Beter Leven (dierenbescherming.nl) in the Netherlands and Bedre Dyrevelferd ((foedevarestyrelsen.dk) in Denmark) as well as in North America. Furthermore, the EU provides organic regulations for animal production, which requires a higher level of animal welfare (e.g. providing access to the range or more space). The initiatives for improving farm animal welfare are multifaceted, involving both international and domestic public policy matters. These initiatives must address scientific, ethical, and economic factors, as well as religious, cultural, and international trade policy aspects (Bayvel & Cross 2010).

The growing association of animal and human health is also a public matter. The majority, 60–75%, of newly emerging infectious diseases have an animal origin (Jones *et al.* 2008): more than a million deaths have been attributable to antimicrobial resistance; and about 3.5 million have been associated with antimicrobial resistance (Murray *et al.* 2022). Impaired farm animal welfare is one of the factors contributing to the risk of antimicrobial resistance. Improvements in farming systems and animals’ living conditions could reduce diseases and therefore the use of antimicrobials. According to the Federation of Veterinarians of Europe (FVE), animals receiving proper care and housing are less likely to develop infections and thus need fewer antibiotics. Enhancing animal welfare is therefore an important approach to lowering antimicrobial use and combating resistance (FVE 2016). This could lead to better results regarding human health, linked with the *One Health, One Welfare* goal (Alonso *et al.* 2020). Alonso *et al.* (2020) called for the supply chain to be informed of public preferences to improve their social responsibility and sustainability. While public preferences may not be expert-based, they reflect societal values and consumer behaviour, which are important considerations for supply chain actors aiming to improve sustainability. Aligning practices with public expectations can enhance social acceptability and support policy development, especially when combined with scientific and technical knowledge.

The OW approach requires cooperation and governance on multiple levels between farmers, other actors in the food processing chain, animal and human health professionals, research, and the authorities, as well as communication with consumers. Farmers’ role is crucial in improving animal welfare. Based on previous studies, farmer well-being is positively associated with farm animal welfare (Hansen & Østerås 2019), while animal welfare is also connected with farm efficiency (Allendorf &Wetteman 2015). Furthermore, Kauppinen *et al.* (2013) showed that farmer well-being was the most important way to improve animal welfare. According to their results, farmers’ motivation for reaching higher animal welfare could be either reward-seeking or empathy. Yet the motivation type makes no difference to improvements in animal welfare or production.

However, the academic literature, especially concerning the One Welfare construct, remains relatively limited. For example, there is little information about how various supply chain actors, including citizens, farmers, and other actors in the food value chain, are aware of and familiar with the OW construct. While previous studies have examined individual dimensions related to OW, such as animal welfare, environmental sustainability, or human well-being, these perspectives are rarely integrated empirically. In particular, there is a lack of empirical research examining how various actors, such as citizens and supply chain stakeholders, cognitively structure the OW construct as a whole. Our study aims to fill the gap in the existing literature and examine how citizens and other supply chain actors perceive the OW construct in connection with improved farm animal welfare. We also aim to compare the views among different European countries. Survey methodology and multivariate methods are adopted to achieve these aims.

## Materials and methods

The citizen data for this study were collected in February 2021, and supply chain stakeholder data in June–July 2021, via online surveys in nine European countries (i.e. Finland, the UK, France, Italy, Belgium, Germany, the Netherlands, Romania, and Denmark) participating in the PPILOW project funded by the European Union (grant agreement N°816172). The stakeholder survey was disseminated to the target population through email distribution lists, newsletters, and farmer and industry associations, drawing on the established national networks of consortium partners. To enhance response rates, multiple reminder messages were circulated during June and July 2021. The stakeholder questionnaire was also available as a paper copy, and this option was used in parallel with the online survey in Romania. The purpose of the data collection was to investigate people’s perceptions of new approaches to pig and poultry production and measures related to farm animal welfare. The questionnaires included themes related to the consumption of and purchase decisions for pork, chicken, and egg products, perceptions of various farming methods, and issues related to animal welfare.

The first survey was targeted at citizens and received a total of 3,601 responses from nine European countries, while the second was directed at supply chain actors (i.e. livestock farmers, livestock farm workers, advisors or consultants, veterinarians, suppliers of animal genetics to farms, slaughterhouses, egg packaging or food processing workers, livestock farming transport workers, retailers, and farmer or industry associations) and received a total of 222 responses.

Our study utilises the question related to animal welfare and especially the One Welfare concept (Table 1). In both questionnaires, OW, connected with improved farm animal welfare, was measured through the aspects of improved human well-being, farming productivity and profitability, food safety and availability, and the environment, based on the existing literature on the concept (e.g. García Pinillos *et al.* 2016). In the questionnaires, respondents were asked to indicate the extent to which improved farm animal welfare (an animal’s experience of its own mental and physical state) is related to various factors. Both citizen and supply chain actor questionnaires contained identical variables related to OW. An exception was that a variable addressing farming profitability was included exclusively in the supply chain actors’ survey due to actors’ ability to assess it based on their expertise and experience, financial ties, and market understanding. Statements were measured on a six-point Likert scale (1 = strongly unrelated, 2 = slightly unrelated, 3 = neither related nor unrelated, 4 = slightly related, 5 = strongly related, 6 = I don’t know). Information about the statements presented in the questionnaire is presented in Table 1.

**Table 1.** Statements included in the citizen and supply chain actor questionnaires to measure respondents’ perceptions of the One Welfare construct based on survey data Table 1. long description.

One welfare: Please indicate to what extent improved farm animal welfare (an animal’s experience of its own mental and physical state) is related to the following aspects.
Improved mental health of humans
Improved physical health of humans
Improved farmer well-being
Improved well-being of people
Improved farming productivity
Improved success of farmers and farming
Improved farming profitability*
Improved food safety
Improved food availability
Improved nature biodiversity
Improved climatic sustainability

* Included only in the supply chain actors’ questionnaire

Table 2 displays sociodemographic information about citizen and supply chain actor samples across different countries, focusing on variables such as gender, average age, and education. Information about the actors’ professions and work experience in the livestock sector is also presented. It should be noted that the sample of supply chain actors from Denmark was too small (n = 3) to allow for meaningful statistical analysis and was therefore excluded from further analyses, whereas the Danish consumer data (n = 400) was retained. Therefore, the sample size of the supply chain actor data in subsequent analyses was 219.

**Table 2.** Sociodemographic background of respondents participating in the survey, by country and respondent group (citizens; n = 3,601, supply chain actors; n = 219) Table 2. long description.

Citizen data (n = 3,601)	Country^1^								
	FI	UK	FR	IT	BE	DE	NL	RO	DK
*N*	400	400	400	400	400	400	400	401	400
*Gender (%)*									
Female	49.1	49.0	50.0	51.0	50.3	50.6	50.2	50.0	50.6
Male	50.9	51.0	50.0	49.0	49.7	49.4	49.8	50.0	49.4
*Age (years, mean)*	50.14	44.50	44.12	42.96	44.04	45.61	44.58	41.91	48.46
*Education (%)*									
Primary education or lower	8.5	4.0	2.3	1.0	2.5	2.8	3.0	1.7	9.8
Secondary education (e.g. high school, vocational education)	33.5	41.0	36.0	9.5	41.8	54.8	50.0	25.7	35.3
Post-secondary education, including bachelor’s degree or equivalent	36.3	40.5.	45.5	54.8	41.0	28.2	35.5	47.9	38.5
Master’s degree or equivalent, or doctorate or equivalent	21.8	14.5	16.3	34.8	14.8	14.2	11.5	24.7	16.5

Supply chain actor data (n = 219)	FI	UK	FR	IT	BE	DE	NL	RO	DK
*N*	29	27	24	39	24	13	16	47	N/A*
*Gender (%)*									
Female	69.0	22.2	41.7	25.6	41.7	61.5	18.8	34.0	N/A*
Male	31.0	74.1	54.2	61.5	58.3	38.5	68.8	63.8	N/A*
No response	–	3.7	–	7.7	–	–	–	2.1	N/A*
Other	–	–	4.2	5.1	–	–	6.3	–	N/A*
*Age (years, mean)*	48.97	48.89	41.63	50.41	46.21	38.85	48.62	43.91	N/A*
*Education (%)*									
Primary education or lower	–	–	–	2.6	4.2	–	–	2.1	N/A*
Secondary education (e.g. high school, vocational education)	24.1	11.1	12.5	5.1	16.7	30.8	50.0	–	N/A*
Post-secondary education, including bachelor’s degree or equivalent	24.1	48.1	58.3	48.7	41.7	15.4	25.0	14.9	N/A*
Master’s degree or equivalent, or doctorate or equivalent	51.7	40.7	29.2	43.6	37.5	53.8	25.0	83.0	N/A*
*Group (%)*									
Livestock farmers or working on a livestock farm	44.8	14.8	50.0	61.6	62.5	46.2	87.5	6.4	N/A*
Other actors^2^	55.1	85.1	50.0	38.5	37.6	53.9	12.5	93.6	N/A*
*Work experience in livestock sector (%)*									
Less than 5 years	3.4	–	20.8	15.4	8.3	7.7	6.3	25.5	N/A*
5–10 years	6.9	14.8	12.5	20.5	20.8	46.2	12.5	14.9	N/A*
10 years or more	89.7	85.2	66.7	64.1	70.8	46.2	81.3	59.6	N/A*

1 FI = Finland, UK = United Kingdom, FR = France, IT = Italy, BE = Belgium, DE = Germany, NL = the Netherlands, RO = Romania, DK = Denmark.

2 Including advisors or consultants, veterinarians, suppliers of animal genetics to farms, slaughterhouses, egg packaging, food processing, livestock farming transports, retailers, farmer or industry associations, other.

* Non applicable. The sample of supply chain actors from Denmark was too small (n = 3) to allow for meaningful statistical analysis and was therefore excluded from further analyses.

To explore the perceptions of the OW construct from citizens’ and other actors’ perspectives, we conducted an exploratory factor analysis (EFA). The selection of items and the interpretation of emerging factors were guided by the conceptual structure of OW that comprises three interrelated domains (i.e. animal welfare, human well-being and environmental sustainability) (e.g. García Pinillos *et al.* 2016). This provided the theoretical basis for expecting that respondents might distinguish between animal-focused, human-focused, and broader sustainability-related elements when evaluating the OW construct. As this has not been explored earlier, EFA was used to identify how these theoretically derived elements are organised in respondents’ perceptions.

In our study, EFA was conducted with Kaiser normalisation, Maximum Likelihood Estimation, and Varimax rotation. As EFA is a multivariate analysis technique designed to reduce the size of a dataset by condensing variables into a smaller number of underlying dimensions, also known as factors or latent variables (Janssens *et al.* 2008), it serves as a valuable tool for identifying groups of attributes. Although maximum likelihood estimation assumes continuous indicators, it is commonly applied to Likert-type data (e.g. Rhemtulla *et al.* 2012). Prior to the EFA, the distributions of all Likert‑type items were examined separately for the citizen and supply chain actor datasets. Skewness values were within acceptable ranges and did not indicate severe departures from normality in either dataset. Therefore, maximum likelihood estimation was considered appropriate. Furthermore, to assess the robustness of our results, we examined a polychoric correlation matrix using the psych package in R, which produced the same factor structure as the maximum likelihood-based solution.

In the analysis, the “I don’t know” responses were considered missing values because they did not contain useful information about respondents’ views of variables related to OW. Furthermore, items with substantial cross loadings (> 0.40 on more than one factor) were removed to ensure a clear and interpretable factor structure. The model adequacy was evaluated using standard EFA diagnostics, including KMO, Bartlett’s test, and scree plot analysis.

In the second stage of analysis, the non-parametric Kruskal-Wallis test was employed, which is similar to ANOVA for three or more independent samples (Fahoome 2002). The Kruskal-Wallis test was conducted to explore the statistically significant differences in the evaluations of the OW factors between the European countries. A non-parametric test was selected because the data did not follow a normal distribution (Fahoome 2002).

To control for Type I error (i.e. the probability of incorrectly rejecting the null hypothesis, see, e.g. Armstrong 2014) due to multiple comparisons, we performed *post hoc* pairwise comparisons using the Bonferroni correction. Although alternative methods, such as the Holm-Bonferroni method, provide a slightly less conservative adjustment, we chose the classical Bonferroni correction to maintain a strict control over false positives. This adjustment ensures that the overall significance level is maintained when conducting multiple tests between pairs of countries. Only differences that remained significant after applying the Bonferroni correction were interpreted as statistically significant. In the statistical analysis, as evidence of the statistical significance of the analysis results, the threshold values were 0.01 ≤ *P* < 0.05 = moderate evidence of statistical significance, and *P* ˂ 0.01 = very strong evidence of statistical significance.

Furthermore, the averages of factor scores in revealed OW factors were calculated separately for each country using the EFA results. Factor scores were standardised (mean = 0), meaning that positive values indicate that a country scored above the overall average on a given factor, while negative values indicate scores below the average. The scores therefore represent relative rather than absolute associations with each latent OW factor. The data were analysed using IBM SPSS® Statistics version 25.0, and supplementary analyses based on polychoric correlations were conducted in R using the psych package.

## Results

### Descriptive statistics

Table 3 presents the evaluations of the OW-related variables by citizens and supply chain actors. For citizen respondents, *improved food safety* was the variable most connected with the OW construct, while for supply chain respondents, the most important linked variable was *improved farmer well-being.* The least connected variable for citizen respondents was *improved mental health of humans*, while for supply chain respondents, the least associated variable was *improved food availability.* The proportion of respondents who answered “I don’t know” was higher among citizens than supply chain actors.

**Table 3.** The variables addressed in the questionnaire and the proportions of respondents by group (i.e. citizens; n = 3,601, supply chain actors; n = 219), according to respondents’ views on the relationship of these variables with the One Welfare construct connected with improved farm animal welfare based on survey responses. The least related variables are underlined; the most related variables are in bold in the table Table 3. long description.

Variable	Strongly unrelated	Slightly unrelated	Neither related nor unrelated	Slightly related	Strongly related	I don’t know %	Averageindex^1^
*Citizens*							
Improved mental health of humans	3.6	4.6	21.3	27.9	25.3	17.3	3.81
Improved physical health of humans	2.9	4.9	19.7	29.8	26.2	16.5	3.85
Improved farmer well-being	2.0	3.0	18.6	32.9	27.4	16.1	3.96
Improved well-being of people	2.8	3.6	19.6	32.5	25.4	16.0	3.88
Improved farming productivity	2.1	6.4	20.3	30.0	24.8	16.4	3.82
Improved success of farmers and farming	1.6	4.6	17.9	32.8	27.1	15.9	3.94
Improved food safety	1.2	2.6	14.7	32.0	36.1	13.5	**4.15**
Improved food availability	2.0	5.1	21.4	31.0	23.8	16.7	3.83
Improved nature biodiversity	1.6	2.9	16.6	31.0	31.0	17.0	4.05
Improved climatic sustainability	2.1	3.7	18.2	30.7	27.7	17.6	3.95
*Supply chain actors*							
Improved mental health of humans	5.0	2.7	16.4	30.1	38.4	7.3	4.01
Improved physical health of humans	6.4	3.2	21.0	33.3	30.1	5.9	3.83
Improved farmer well-being	3.2	2.3	15.1	27.9	46.6	5.0	**4.18**
Improved well-being of people	5.9	2.3	15.5	36.5	31.5	8.2	3.93
Improved farming productivity	7.8	11.0	18.7	32.9	25.1	4.6	3.59
Improved success of farmers and farming	4.6	3.2	16.0	34.2	35.6	6.4	4.00
Improved farming profitability	6.4	6.4	20.1	33.8	28.3	5.0	3.75
Improved food safety	7.3	5.5	15.5	30.1	36.5	5.0	3.88
Improved food availability	11.0	9.6	22.4	23.7	26.9	6.4	3.49
Improved nature biodiversity	5.9	4.1	15.5	32.0	36.1	6.4	3.94
Improved climatic sustainability	10.0	7.3	16.0	29.7	27.4	9.6	3.63

1 The last column represents an index that was obtained by calculating the average of responses by each variable (scale 1–5). The average index was calculated by excluding the ‘I don’t know’ responses.

### Factor solutions for citizens’ and supply chain actors’ views of One Welfare

**Figure 1.**
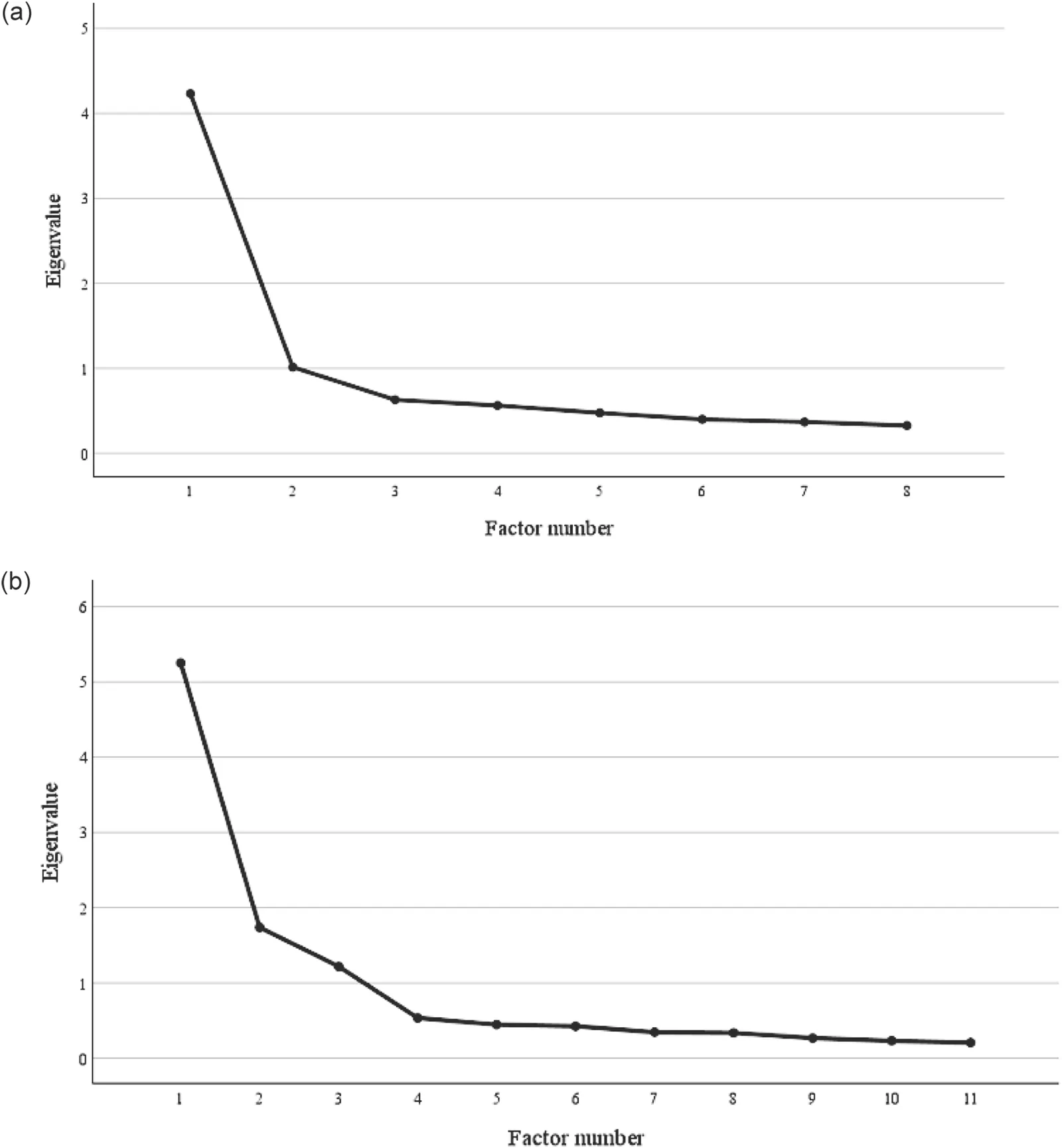
Scree plot of initial eigenvalues from (a) the citizen dataset (n = 3,601) based on survey data. The plot shows a clear elbow after the second factor, with all subsequent eigenvalues falling below 1.0, supporting the retention of a two‑factor solution. Scree plot of initial eigenvalues from (b) the supply chain actor dataset (n = 219) based on survey data. Although the largest decline occurs after the first factor, the second and third eigenvalues exceed 1.0, supporting the retention of a three‑factor solution.

First, EFA was conducted to explore the perceptions of the OW construct connected with improved farm animal welfare from citizens’ perspectives, and it resulted in a two-factor solution. To determine the appropriate number of factors, we examined the scree plots based on the initial eigenvalues. In the citizen dataset (Figure 1[a]), the first eigenvalue was significantly larger (4.23) than the second (1.01), after which the curve levelled off and all remaining eigenvalues fell below 1.0. This pattern is characteristic of a two‑factor solution. The final EFA solution consisted of 8 variables out of 10 used in the questionnaire, and the solution explained approximately 55% of our data’s total variance (Table 4). During the EFA modelling, the variables related to improved natural biodiversity and climate sustainability were omitted from the final factor solution due to cross-loadings. The Kaiser-Meyer-Olkin measure of factorability for the results was 0.888, ensuring the data were suitable for use in EFA. Bartlett’s test of sphericity rejected the null hypothesis that no correlation among the original variables existed (*P* = 0.000).

**Table 4.** Results of the final rotated two‑factor exploratory factor analysis solution for variables describing citizens’ (n = 3,601) perceptions of One Welfare based on survey data. The two extracted factors were labelled as health and well‑being and farming success and food security, reflecting key conceptual domains of the One Welfare framework. Bold values indicate the highest absolute factor loadings Table 4. long description.

Variables	Communalities (extracted)	F1: Health and well-being	F2: Farming success andfood security
Improved mental health of humans	0.557	**0.689**	0.288
Improved physical health of humans	0.695	**0.799**	0.238
Improved farmer well-being	0.496	**0.565**	0.420
Improved well-being of people	0.642	**0.743**	0.299
Improved farming productivity	0.515	0.227	**0.681**
Improved success of farmers and farming	0.645	0.224	**0.771**
Improved food safety	0.437	0.391	**0.533**
Improved food availability	0.406	0.374	**0.516**
*Cronbach’s alpha*		0.861	0.805
*Eigenvalues*		4.227	1.013
*Explained variance (total 55%)*		29.740	25.160

EFA results revealed two underlying dimensions of the OW construct: Factor 1, ‘Health and well-being’; and Factor 2, ‘Farming success and food security’. The first factor, ‘Health and well-being’, consists of the improved mental and physical health of humans, and improved well-being of farmers and people, variables, while the second factor, ‘Farming success and food security’, includes the variables regarding improved food safety and availability, and the variables related to improved farming productivity and improved success of farmers and farming.

Second, we examined the OW construct from other supply chain actors’ perspective. In contrast to the citizen dataset, the scree plot for the supply chain actor data (Figure 1[b]) suggested a more complex factor structure. Although the steepest decline occurred after the first eigenvalue (5.25), both the second and third eigenvalues exceeded 1.0, supporting the retention of three factors. Consequently, EFA resulted in a three-factor solution. The final EFA solution consisted of 11 variables used in the questionnaire, and the solution explained approximately 65% of the total variance of our data (Table 5). The Kaiser-Meyer-Olkin measure of factorability for the results was 0.859, ensuring the data were suitable for use in EFA. Bartlett’s test of sphericity rejected the null hypothesis that no correlation among the original variables existed (*P* = 0.000).

**Table 5.** Results of the final rotated three‑factor exploratory factor analysis solution for variables describing supply chain actors’ (n = 219) perceptions of One Welfare based on survey data. The three extracted factors were labelled as health and well‑being, food and nature sustainability, and profitability and success, corresponding to core domains discussed in the One Welfare literature. Bold values indicate the highest absolute factor loadings Table 5. long description.

Variables	Communalities (extracted)	F1: Health and well-being	F2: Food and nature sustainability	F3: Profitability and success
Improved mental health of humans	0.635	**0.775**	0.119	0.143
Improved physical health of humans	0.673	**0.730**	0.367	0.066
Improved farmer well-being	0.656	**0.750**	0.095	0.291
Improved well-being of people	0.573	**0.658**	0.367	0.072
Improved farming productivity	0.603	0.167	0.172	**0.739**
Improved farming profitability	0.825	0.076	0.191	**0.885**
Improved success of farmers and farming	0.648	0.237	0.295	**0.710**
Improved food safety	0.631	0.145	**0.715**	0.313
Improved food availability	0.634	0.093	**0.672**	0.417
Improved nature biodiversity	0.607	0.383	**0.669**	0.114
Improved climatic sustainability	0.699	0.340	**0.747**	0.157
*Cronbach’s alpha*		0.873	0.871	0.859
*Eigenvalues*		5.246	1.737	1.219
*Explained variance (total 65%)*		22.828	21.960	20.515

EFA results revealed three underlying dimensions of the OW construct: Factor 1, ‘Health and well-being’; Factor 2, ‘Food and nature sustainability’; and Factor 3 ‘Profitability and success’. The first factor, ‘Health and well-being’, included variables of improved mental and physical health of humans, and improved well-being of farmers and people. The second, ‘Food and nature sustainability’, consisted of improved sustainability related to nature and climate, and the variables regarding improved food safety and availability. Finally, the third, ‘Profitability and success’, comprised variables related to economic aspects, including improved farming productivity and profitability, and improved success of farmers and farming.

### Differences in One Welfare views among European countries

The differences between the OW perceptions in different European countries were analysed with the Kruskal-Wallis test and by calculating average factor scores for the revealed EFA factors by country.

#### Kruskal-Wallis test results

The Kruskal-Wallis test was conducted to explore the differences in OW views among European countries. First, citizens’ perceptions were examined. Table 6 presents the Kruskal-Wallis test results for statistically significant differences in citizens’ perceptions of OW dimensions by country. Our results show that statistically significant differences exist between citizens living in different European countries in their evaluations of ‘Health and well-being’ (Factor 1) and ‘Farming success and food security’ (Factor 2) (Table 6).

**Table 6.** Results of Kruskal–Wallis tests comparing citizens’ perceptions of One Welfare dimensions (factors derived from exploratory factor analysis) across countries based on survey data Table 6. long description.

Factor	Groups compared	*P-*value for all groups	*P*-value for groupwise comparisons^a^
Health and well-being	All countries	< 0.001***	
	Italy vs Finland		0.001***
	Italy vs Denmark		0.001***
	Italy vs the United Kingdom		0.010**
	Italy vs Belgium		0.015**
	Italy vs the Netherlands		0.002***
Farming success and food security	All countries	< 0.001***	
	Italy vs the United Kingdom		0.021**
	Italy vs Belgium		0.001***
	France vs Italy		0.000***
	France vs The Netherlands		0.000***
	France vs Denmark		0.002***
	France vs Germany		0.009***
	France vs Finland		0.011**
	Romania vs The Netherlands		0.000***
	Romania vs Italy		0.000***
	Romania vs Denmark		0.002***
	Romania vs Germany		0.008***
	Romania vs Finland		0.010**

a Significance values have been adjusted by the Bonferroni correction for multiple tests.

**Moderate evidence of statistical significance = 0.01 ≤ *P* < 0.05; ***Very strong evidence of statistical significance = *P* ˂ 0.01.

In ‘Health and well-being’ (Factor 1 for citizens), Italian respondents differed statistically significantly from other countries (*P*-values ranged from 0.001 to 0.015), such as Finland, Denmark, the UK, Belgium and the Netherlands. In the case of citizens’ perceptions of the second factor, ‘Farming success and food security’, there were multiple statistically significant differences between countries. For example, French respondents differed statistically significantly from several other countries (*P*-values ranged from 0.000 to 0.011). Romanian respondents also differed from others (*P*-values ranged from 0.000 to 0.010). Furthermore, Italy differed from four countries (*P*-values ranged from 0.000 to 0.021), including the UK, Romania, France, and Belgium.

Second, we investigated the supply chain actors’ views of the three revealed factors (Table 7). Our results provided evidence of statistically significant differences among certain countries in terms of ‘Food and nature sustainability’ (Factor 2) and ‘Profitability and success’ (Factor 3). Table 7 presents detailed information about how supply chain actors’ views differed by statistical measures at the level of individual countries.

**Table 7.** Results of Kruskal–Wallis tests comparing supply chain actors’ perceptions of One Welfare dimensions (factors derived from exploratory factor analysis) across countries based on survey data Table 7. long description.

Factor	Groups compared	*P-*value for all groups	*P*-value for groupwise comparisons^a^
Food and nature sustainability	All countries	< 0.001***	
	Italy vs the United Kingdom		0.001***
	Italy vs the Netherlands		0.013**
	Italy vs France		0.019**
	Romania vs Belgium		0.000***
	Romania vs the United Kingdom		0.000***
	Romania vs the Netherlands		0.000***
	Romania vs France		0.000***
	Romania vs Germany		0.003***
	Romania vs Finland		0.000***
Profitability and success	All countries	< 0.001***	
	The Netherlands vs Finland		0.015**
	The Netherlands vs Romania		0.006***
	Italy vs Romania		0.004***
	Italy vs Finland		0.020**

a Significance values have been adjusted by the Bonferroni correction for multiple tests.

**Moderate evidence of statistical significance = 0.01 ≤ *P* < 0.05; ***Very strong evidence of statistical significance = *P* ˂ 0.01.

In the case of supply chain actors’ perceptions of ‘Food and nature sustainability’, Italian and Romanian respondents differed statistically significantly from other countries. For example, Italy differed from three countries (the UK, the Netherlands, France) (*P*-values ranged from 0.001 to 0.019). Romania differed from six countries (Belgium, the UK, the Netherlands, France, Germany, and Finland) (*P*-values ranged from 0.000 to 0.003). For the factor, ‘Profitability and success’, Dutch and Italian respondents differed from two other countries. For example, the Netherlands differed from Finland and Romania (*P*-values ranged from 0.006 to 0.015). Italy also differed from the same countries (Finland and Romania) (*P*-values ranged from 0.004 to 0.020).

#### Average factor scores

Next, we calculated the average factor scores for the revealed EFA factors for both citizens and supply chain actors living in nine European countries. Table 8 presents the detailed information about the average factor scores: a higher average factor score indicates a higher rating for the factor; a lower average score suggests a lower rating. In our study, the higher the average factor score, the more connected the factor was to the OW construct from the respondent’s perspective.

**Table 8.** Average standardized factor scores (mean = 0) of One Welfare dimensions derived from exploratory factor analysis, presented by country and respondent group (citizens and supply chain actors) participating in a survey on perceptions of the One Welfare construct Table 8. long description.

Country^1^	EFA model 1 (citizens)		EFA model 2 (supply chain actors)		
	F1: Health and well-being	F2: Farming success and food security	F1: Health andwell-being	F2: Food and nature sustainability	F3: Profitability and success
FI	–0.085	–0.078	0.308	–0.194	0.329
DK	–0.080	–0.054	N/A*	N/A*	N/A*
RO	0.045	0.125	0.045	0.641	0.345
UK	–0.007	0.055	–0.116	–0.462	0.284
DE	0.039	–0.050	0.213	–0.504	–0.450
BE	–0.039	0.067	–0.291	–0.223	–0.026
NL	–0.030	–0.077	–0.331	–0.400	–0.488
FR	–0.027	0.160	–0.037	–0.373	–0.200
IT	0.184	–0.148	0.063	0.392	–0.368

1 FI = Finland, UK = United Kingdom, FR = France, IT = Italy, BE = Belgium, DE = Germany, NL = Netherlands, RO = Romania, DK = Denmark.

* Non applicable. The sample of supply chain actors from Denmark was too small (n = 3) to allow for meaningful statistical analysis and was therefore excluded from further analyses.

**Figure 2.**
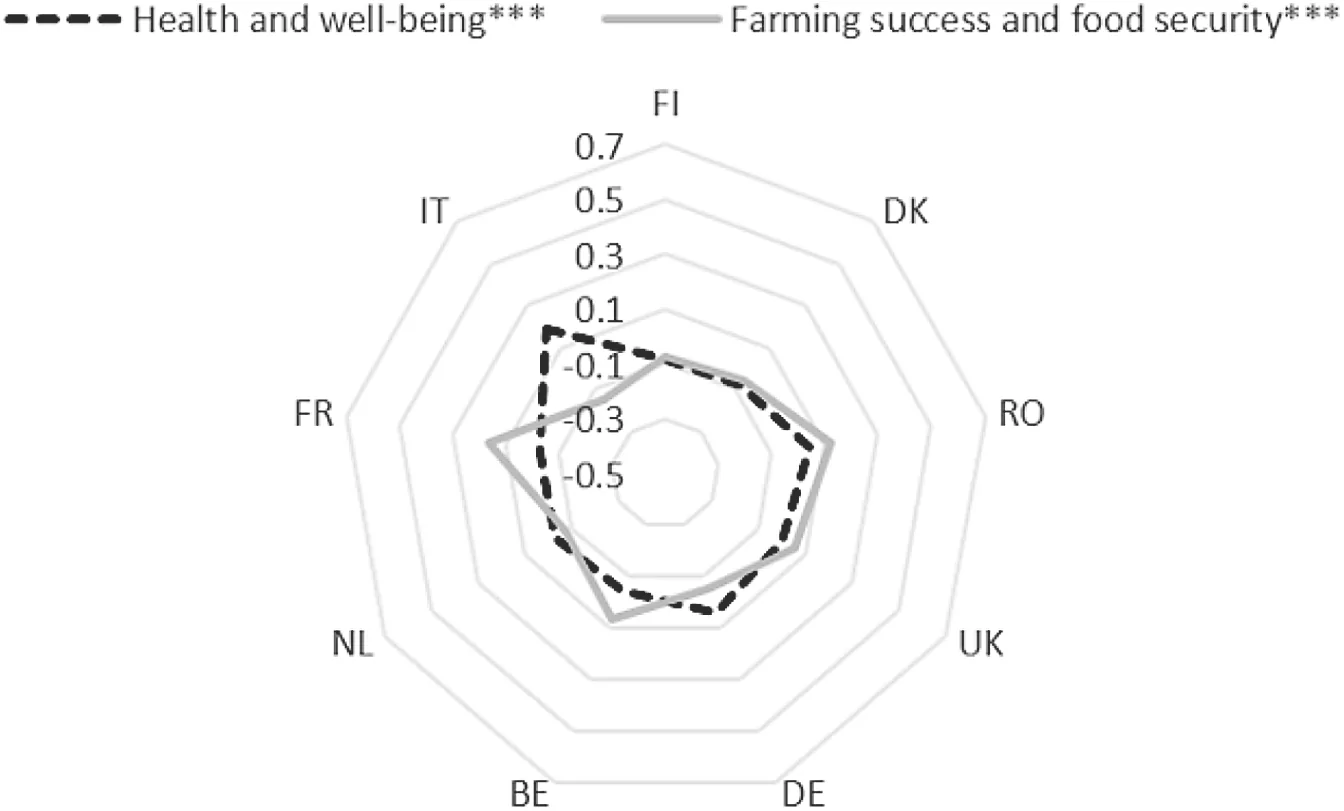
Average factor scores of the two One Welfare dimensions derived from exploratory factor analysis, presented by country^1^ for citizens (n = 3,601) participating in a survey on perceptions of the One Welfare construct. The figure illustrates country‑level differences in how citizens associate One Welfare with health and well‑being and with farming success and food security. ^1^FI = Finland, UK = United Kingdom, FR = France, IT = Italy, BE = Belgium, DE = Germany, NL = the Netherlands, RO = Romania, DK = Denmark.

**Figure 3.**
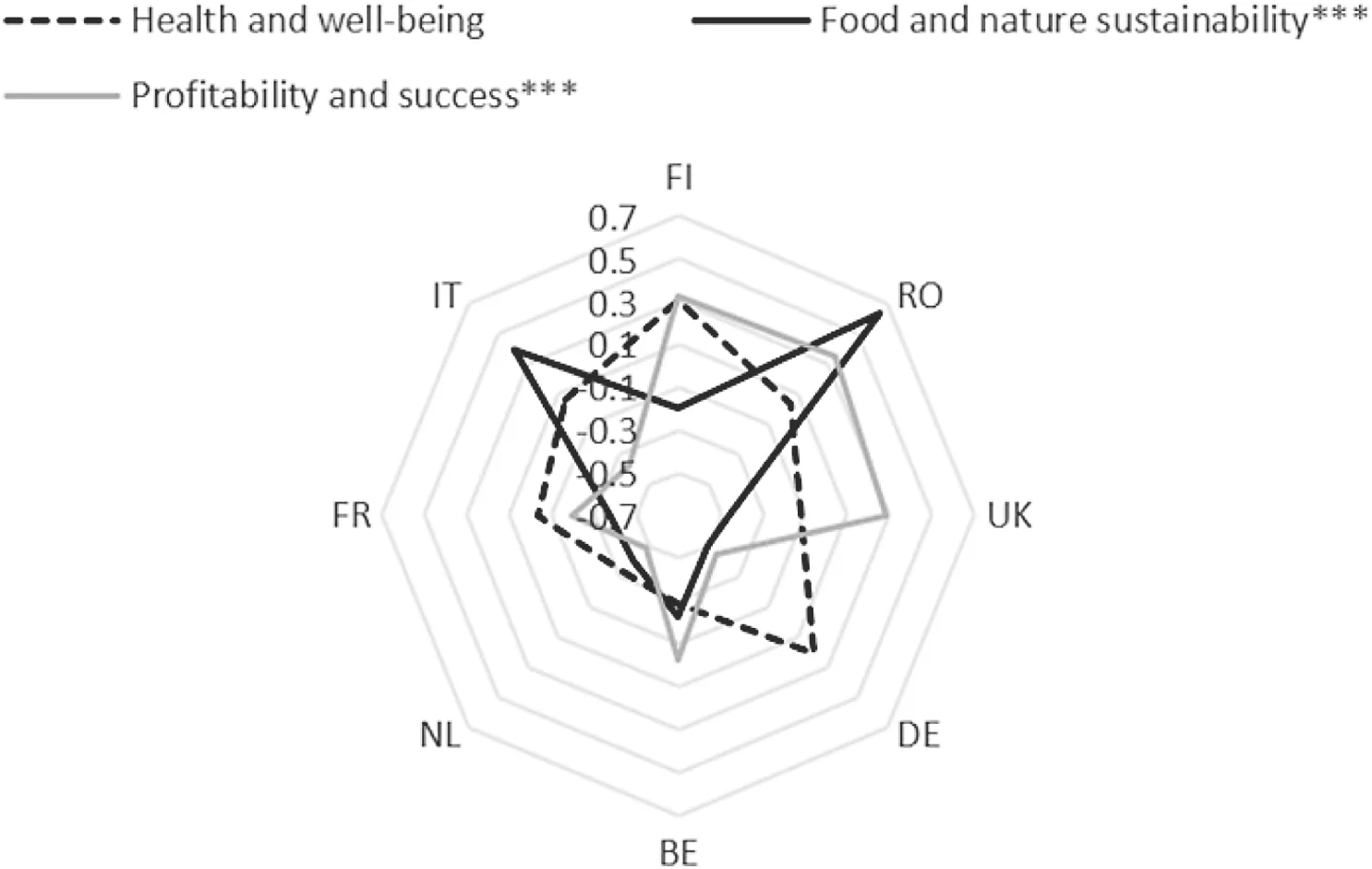
Average factor scores of the three One Welfare dimensions derived from exploratory factor analysis, presented by country^1^ for supply chain actors (n = 219) participating in a survey on perceptions of the One Welfare construct. The figure illustrates country‑level differences in how supply chain actors associate One Welfare with health and well‑being, food and nature sustainability, and profitability and success. ^1^FI = Finland, UK = United Kingdom, FR = France, IT = Italy, BE = Belgium, DE = Germany, NL = the Netherlands, RO = Romania.

The radar charts (Figures 2 and 3) visualise country level deviations from the mean factor scores. Lines extending further from the centre indicate relatively stronger associations with the factor, whereas lines closer to the centre indicate weaker associations. These visual differences reflect relative positioning across countries. For citizens and based on the average factor scores, the two revealed factors had quite similar evaluations across countries (Figure 2). In Italy, Romania, and Germany, the evaluations of the factor ‘Health and well-being’ were higher than other countries (average factor scores 0.039–0.184). For the ‘Farming success and food security’ factor, respondents from Romania, France, Belgium, and the UK also gave higher ratings (average factor scores 0.055–0.160) than respondents from Italy, Finland, Denmark, Germany, and the Netherlands (average factor scores ranging from –0.148 to –0.050). Furthermore, Italian, Dutch, and German respondents considered the factor ‘Health and well-being’ to be more connected with the OW construct than ‘Farming success and food security’. In contrast, respondents from France, Finland, Denmark, Romania, the UK, Belgium, considered ‘Farming success and food security’ as more closely associated with the construct than ‘Health and well-being’. However, the Kruskal-Wallis test results indicated that statistically significant differences emerged only for certain countries and factor evaluations, for instance among Italy, Romania, and France.

For supply chain actors, based on average factor scores, there were stronger differences between the countries and the factor evaluations compared to citizens (Figure 3). For example, in terms of the ‘Health and well-being’ factor, respondents from the Netherlands gave the lowest ratings (average factor score –0.331) and therefore differed from several other countries. However, based on the Kruskal-Wallis test results, these differences were not statistically significant. For the second factor, ‘Food and nature sustainability’, respondents from Romania and Italy gave the highest ratings (average factor scores 0.392–0.641). For ‘Profitability and success’, there were statistically significant differences between the Netherlands and other countries. While respondents from the Netherlands gave the lowest ratings (average factor score –0.488), respondents from Romania gave the highest ratings (average factor score 0.345).

Furthermore, supply chain respondents from Finland, Belgium, and the UK considered ‘Profitability and success’ to be the most connected factor with the OW construct. However, in Romania and Italy, respondents considered ‘Food and nature sustainability’ to be the most linked factor with the construct. ‘Health and well-being’ was the most associated factor with the OW construct in France, the Netherlands, and Germany. In general, the average factor scores for all factors were larger among supply chain than citizen respondents.

## Discussion

The purpose of this study was to examine how citizens and other actors perceived the OW construct in connection with improved farm animal welfare and to compare the views among different European countries. Our results showed that citizens and supply chain actors had differing views of the variables related to improved farm animal welfare and the OW construct. For example, improved food safety was the most connected variable with the construct, while human mental health was the least related variable from citizens’ perspective. From supply chain actors’ perspective, the most associated variable with improved farm animal welfare and the OW construct was improved farmer well-being, while the least connected variable was improved food availability. However, it is important to note that the average indexes of all variables were quite close to each other.

COVID-19 increased consumers’ awareness of antimicrobial resistance and the connection between animal and human health (Regan *et al.* 2021), which supports the association of animal welfare and OW with food safety indicated by our study. Furthermore, one explanation for the close relationship of farmer well-being to animal welfare and the OW construct from the supply chain actors’ perspective might be that veterinarians are in a position to identify mental health struggles in farmers as their clients, and animal welfare and behaviour can show clues of this (Ritter & King 2023).

Additionally, according to our results, supply chain actors have a more multidimensional understanding of animal welfare and the OW construct. Although OW seems to be a two-dimensional construct for citizens, consisting mainly of aspects related to human well-being and farming success and food security, for supply chain actors, food and nature sustainability is also a distinct dimension of OW. This issue may be due to supply chain actors’ deeper awareness of animal production, practices, food production, and their impacts on the environment, animals, and humans. This is also supported by the fact that the proportion of citizens in general who answered “I don’t know” in our data was larger than the proportion of supply chain actors responding “I don’t know”. The high share of “I don’t know” responses among citizens likely reflects limited familiarity with the OW construct. OW’s interdisciplinary nature, linking human, animal, and environmental welfare, may be difficult to understand without prior exposure. In particular, the connection between animal welfare and human mental health may be less intuitive. These findings highlight the need for clearer public communication on OW.

Previous studies also support this interpretation: Chiesa *et al.* (2021) found that even experts struggled to define the related One Health (OH) concept. As stated in the *Introduction*, OH is a similar concept to OW, addressing the interconnected approaches to health, emphasising the commonalities of human, animal, plant, and environmental health (Radeski *et al.* 2018). According to Chiesa *et al.* (2021), more than half the experts (mainly from academic research, but also from ministries, the private sector, and NGOs, associations, and scientific societies) declared they had heard about the OH concept, even though only about a tenth of respondents were able to define OH properly.

Additionally, Hempel *et al.* (2023) showed that consumers often lack knowledge of animal production practices. This low level of knowledge complicates the ability to distinguish the effects of animal and food production on environment, animal welfare, producers, health, and society, which is also a barrier to more sustainable consumer behaviour. Previous studies have also indicated that health and environmental aspects have a stronger effect on the consumption behaviour than animal welfare (e.g. Grummon *et al.* 2023).

Furthermore, our results showed that statistically significant differences existed between certain European countries. For example, Italy and Romania seem to show statistically significant differences compared to other countries. For Italian citizens, improved farm animal welfare and OW are most closely tied to human well-being, while Romanian citizens link them to farming success and food security. Yet both Italian and Romanian supply chain actors agree that food security and environmental sustainability are the most associated aspects with improved farm animal welfare and OW.

These results may be explained partly by the characteristics of the countries’ production; Italy is one of the leading countries in terms of organic production (Agence BIO 2021), whereas Romania has a strong history of family farming (FAO 2024). Furthermore, in Italy, health issues have been the most important reason for purchasing organic products (Agence BIO 2021; p 24), and our results also indicated that Italian respondents considered the factor related to health and well-being to be most connected with improved farm animal welfare and the OW construct, which may reflect the importance of health issues for Italians. The country-level differences may also reflect broader cultural contexts of animal welfare and food systems. Societal values, agricultural traditions, and public discourse around sustainability and ethics can influence how the OW dimensions are perceived. In this sense, socio-political priorities and economic conditions likely contribute to why certain aspects of OW, such as health, food security, or environmental sustainability, are emphasised differently across contexts.

Notably, French citizens differed significantly from the others in their evaluation of the farming success and food security factor, associating it more strongly with the OW construct and improved farm animal welfare than with health and well-being aspects. This may reflect France’s strong cultural and historical connection to agriculture, as well as widespread public awareness of food security and farming issues (Rogers 2000). Furthermore, the National Pathway toward Sustainable Food Systems in France emphasises farm economics and income as one of the key priorities (UN Food Systems Hub 2023), which may help explain why French citizens perceive improved farm animal welfare and OW as more directly linked to farming outcomes, such as improved farming productivity and improved success of farmers and farming, while health and well-being aspects are seen as more general or indirect.

### Study limitations

A significant sampling limitation of this study is that the response rate of the stakeholder survey was limited (e.g. there were only three respondents from Denmark and therefore the country was not included in the further analysis), and the respondents did not include representatives from all the stakeholder groups that the survey intended to reach in each country. Due to these sampling constraints, it was not meaningful to conduct a deeper analysis related to different professional groups such as farmers. Another point of caution is that the responses of supply chain actors may not be representative of the sector as a whole. One must therefore be cautious in generalising the results for supply chain actors. In future studies, perceptions of different stakeholder groups could be investigated further, which could bring more insights into the phenomena.

Our study also showed methodological limitations. Although our analyses used maximum likelihood estimation, which assumes continuous and normally distributed variables, the items were measured on ordinal Likert scales. However, prior work suggests that maximum likelihood estimation is robust with Likert data when 5-point scales are used and distributions are not highly skewed (Rhemtulla *et al.* 2012). Furthermore, a supplementary analysis using polychoric correlations produced an almost identical factor pattern, indicating that the results are stable across estimation methods.

Additionally, in the analysis, two items (‘improved climatic sustainability’ and ‘improved biodiversity’) were removed from the citizen EFA because they loaded substantially on multiple factors. Their exclusion therefore improved the clarity and discriminant validity of the factor solution. Although these issues are theoretically integral to the OW construct and literature, in our citizen dataset, they did not operate as distinct factors. The removal of these items reflects conceptual differences in how our respondents, citizens and other supply chain actors, understand the OW construct. Citizens appear to treat biodiversity and climatic sustainability as broad cross-cutting themes rather than as a specific, isolatable dimension. In contrast, supply chain actors associated these environmental items more directly with food safety and practical sustainability, that formed a coherent factor in that dataset. These differences highlight how OW elements may be perceived in distinct ways across various stakeholders.

Also, the high share of “I don’t know” responses among citizens may reflect both limited familiarity with OW principles and potential ambiguity in the survey items, which could have influenced response consistency and should therefore be considered a limitation. Furthermore, as our study employed only quantitative analysis, qualitative methods such as interviews could also be applied to gain a more comprehensive understanding of the OW construct from different actors’ perspectives.

Besides the country of residence, we did not conduct an analysis of how various sociodemographic background variables (e.g. age, gender, education) influenced the OW perceptions of citizens and other actors. The Kruskal-Wallis test was used as a descriptive, group level comparison, and the absence of covariate adjustment represents a limitation of the present study. Previous studies have shown that age, gender, and education influence how consumers perceive animal welfare-related issues such as the One Health concept (e.g. Wu *et al.* 2023), and these effects may also differ across countries. Accordingly, this highlights an important avenue for future research. To summarise, the data of our study were collected from nine countries, which is a major advantage of the study and allows country-wise comparisons. It is noteworthy that there may be country-wise differences in responding to surveys that should be considered in the interpretation of the results.

### Animal welfare implications

Our results contribute to enhancing the understanding and implementation of the OW approach in Europe. For example, our study is relevant for policymakers, as the results offer information about the current understanding and perception of OW among different European countries. In addition, our results suggest that citizens may not be fully aware of how animal welfare is connected with human health and the environment, and more efficient communication of these issues is therefore required.

Understanding how various supply chain actors, including citizens, farmers, and other actors in the food value chain, perceive OW and animal welfare provides crucial information for policymakers to develop policies and regulations that respond to both citizens’ expectations and industry’s capabilities. This approach allows creating policies that are more acceptable and effective. As OW addresses the interconnectedness of human well-being, animal welfare, and environmental health, policies promoting OW can assist in achieving broader sustainability goals related to, e.g. food security, biodiversity conservation, and public health.

Our results showed, for example, that supply chain actors have a more multifaceted view of OW in relation to improved farm animal welfare than citizens. This is particularly significant from the perspective of animal welfare, as these actors are more likely to interact with animals directly. In contrast, citizens’ limited understanding may influence their willingness to support or advocate for animal welfare.

Animal welfare information could be used to raise citizens’ awareness of the OW approach. Citizens’ awareness of OW should be deepened because COVID-19, for example, brought out the effects of pandemics on food production and animal welfare (Marchant-Forde & Boyle 2020). The pandemic influenced intensive animal production through the temporary closure of processing facilities, which overcrowded the slaughtering, in turn affecting the environment by disposal of carcases. To deepen citizens’ understanding of OW and animal welfare issues, our results could be used to create awareness among the general public by developing educational campaigns and communication strategies. This could help citizens lead more sustainable lifestyles; to increase sustainable consumption habits, citizens need a more comprehensive understanding of OW, including the importance of zoonotic diseases and responsible antibiotic use, which are critical public health issues. Communication also to the supply chain actors would help policymakers by ensuring that these actors understand and support the welfare standards and regulations.

Furthermore, our results provided evidence that the OW construct is perceived differently across European countries both among citizens and other supply chain actors. For example, the differences between Italy and Romania were significant and are partly explained by differences in production methods and traditions, such as organic production and family farming. Therefore, developing policies should be tailored to certain regions and adjusted in terms of local conditions.

## Conclusion

One Welfare emphasises the interconnection of human well-being, animal welfare, and the environment. It is of major importance in improving both animal and human welfare (García Pinillos *et al.* 2016). Despite this, academic research on OW perceptions among different actors has been very limited. Our results indicated that OW was a multidimensional issue for various supply chain actors; however, it was not as multifaceted for citizens. Additionally, our results showed that statistically significant differences existed between certain European countries in how they perceived the dimensions of the OW construct, especially in connection with improved farm animal welfare. Enhancing awareness and understanding of OW among citizens and other actors is crucial for developing policies that promote public health, environmental sustainability, and ethical standards.

## Long descriptions

### Table 1. Long description

The table begins with a header question: One welfare: Please indicate to what extent improved farm animal welfare (an animal’s experience of its own mental and physical state) is related to the following aspects.

The following eleven items are listed vertically:

* Improved mental health of humans

* Improved physical health of humans

* Improved farmer well-being

* Improved well-being of people

* Improved farming productivity

* Improved success of farmers and farming

* Improved farming profitability (marked with an asterisk indicating this was included only in the supply chain actors’ questionnaire)

* Improved food safety

* Improved food availability

* Improved nature biodiversity

* Improved climatic sustainability

Navigate back to Table 1..

### Table 2. Long description

The table is divided into two main sections.

Section 1: Citizen data (n = 3,601).

- Sample size (N): 400 for all countries except R O (401).

- Gender: Female percentages range from 49.0 in U K to 51.0 in I T. Male percentages range from 49.0 in I T to 51.0 in U K.

- Mean Age: Highest in F I (50.14) and lowest in R O (41.91).

- Education: Post-secondary education is the most common level in most countries, peaking at 54.8 percent in I T. Master's or Doctorate degrees range from 11.5 percent in N L to 34.8 percent in I T.

Section 2: Supply chain actor data (n = 219).

- Sample size (N): Ranges from 13 in D E to 47 in R O. D K is excluded due to small sample size.

- Gender: Female representation varies significantly, from 18.8 percent in N L to 69.0 percent in F I.

- Mean Age: Ranges from 38.85 in D E to 50.41 in I T.

- Education: Master's or Doctorate degrees are highly prevalent, reaching 83.0 percent in R O.

- Group: Livestock farmers make up the majority in N L (87.5 percent), while other actors (veterinarians, processors, etc.) dominate in R O (93.6 percent) and U K (85.1 percent).

- Work experience: Most respondents have 10 years or more experience, with the highest proportion in F I (89.7 percent).

Navigate back to Table 2..

### Table 3. Long description

The table contains eight columns: Variable, Strongly unrelated, Slightly unrelated, Neither related nor unrelated, Slightly related, Strongly related, I don’t know %, and Average index (calculated on a 1 to 5 scale).

Citizens Group (n = 3,601):

* Improved mental health of humans: 3.6% strongly unrelated, 4.6% slightly unrelated, 21.3% neither, 27.9% slightly related, 25.3% strongly related, 17.3% don't know. Average index: 3.81 (underlined as least related).

* Improved physical health of humans: Average index 3.85.

* Improved farmer well-being: Average index 3.96.

* Improved well-being of people: Average index 3.88.

* Improved farming productivity: Average index 3.82.

* Improved success of farmers and farming: Average index 3.94.

* Improved food safety: 1.2% strongly unrelated, 2.6% slightly unrelated, 14.7% neither, 32.0% slightly related, 36.1% strongly related, 13.5% don't know. Average index: 4.15 (bolded as most related).

* Improved food availability: Average index 3.83.

* Improved nature biodiversity: Average index 4.05.

* Improved climatic sustainability: Average index 3.95.

Supply chain actors Group (n = 219):

* Improved mental health of humans: Average index 4.01.

* Improved physical health of humans: Average index 3.83.

* Improved farmer well-being: 3.2% strongly unrelated, 2.3% slightly unrelated, 15.1% neither, 27.9% slightly related, 46.6% strongly related, 5.0% don't know. Average index: 4.18 (bolded as most related).

* Improved well-being of people: Average index 3.93.

* Improved farming productivity: Average index 3.59.

* Improved success of farmers and farming: Average index 4.00.

* Improved farming profitability: Average index 3.75.

* Improved food safety: Average index 3.88.

* Improved food availability: 11.0% strongly unrelated, 9.6% slightly unrelated, 22.4% neither, 23.7% slightly related, 26.9% strongly related, 6.4% don't know. Average index: 3.49 (underlined as least related).

* Improved nature biodiversity: Average index 3.94.

* Improved climatic sustainability: Average index 3.63.

Navigate back to Table 3..

### Table 4. Long description

The table consists of four columns: Variables, Communalities (extracted), F 1: Health and well-being, and F 2: Farming success and food security.

Factor 1 (Health and well-being) primary loadings:

* Improved mental health of humans: Communality 0.557, F 1 0.689, F 2 0.288.

* Improved physical health of humans: Communality 0.695, F 1 0.799, F 2 0.238.

* Improved farmer well-being: Communality 0.496, F 1 0.565, F 2 0.420.

* Improved well-being of people: Communality 0.642, F 1 0.743, F 2 0.299.

Factor 2 (Farming success and food security) primary loadings:

* Improved farming productivity: Communality 0.515, F 1 0.227, F 2 0.681.

* Improved success of farmers and farming: Communality 0.645, F 1 0.224, F 2 0.771.

* Improved food safety: Communality 0.437, F 1 0.391, F 2 0.533.

* Improved food availability: Communality 0.406, F 1 0.374, F 2 0.516.

Statistical Summary:

* Cronbach’s alpha: F 1 is 0.861, F 2 is 0.805.

* Eigenvalues: F 1 is 4.227, F 2 is 1.013.

* Explained variance (total 55%): F 1 is 29.740, F 2 is 25.160.

Navigate back to Table 4..

### Table 5. Long description

The table consists of 5 columns: Variables, Communalities (extracted), F 1: Health and well-being, F 2: Food and nature sustainability, and F 3: Profitability and success.

Factor 1 (Health and well-being) variables and loadings:

* Improved mental health of humans: Communality 0.635, F 1 loading 0.775.

* Improved physical health of humans: Communality 0.673, F 1 loading 0.730.

* Improved farmer well-being: Communality 0.656, F 1 loading 0.750.

* Improved well-being of people: Communality 0.573, F 1 loading 0.658.

Factor 2 (Food and nature sustainability) variables and loadings:

* Improved food safety: Communality 0.631, F 2 loading 0.715.

* Improved food availability: Communality 0.634, F 2 loading 0.672.

* Improved nature biodiversity: Communality 0.607, F 2 loading 0.669.

* Improved climatic sustainability: Communality 0.699, F 2 loading 0.747.

Factor 3 (Profitability and success) variables and loadings:

* Improved farming productivity: Communality 0.603, F 3 loading 0.739.

* Improved farming profitability: Communality 0.825, F 3 loading 0.885.

* Improved success of farmers and farming: Communality 0.648, F 3 loading 0.710.

Summary Statistics:

* Cronbach’s alpha: F 1 is 0.873, F 2 is 0.871, F 3 is 0.859.

* Eigenvalues: F 1 is 5.246, F 2 is 1.737, F 3 is 1.219.

* Explained variance (total 65%): F 1 is 22.828, F 2 is 21.960, F 3 is 20.515.

Navigate back to Table 5..

### Table 6. Long description

A table with four columns: Factor, Groups compared, P-value for all groups, and P-value for groupwise comparisons.

* Factor: Health and well-being

- All countries: P-value < 0.001 (very strong significance).

- Italy vs Finland: P-value 0.001 (very strong significance).

- Italy vs Denmark: P-value 0.001 (very strong significance).

- Italy vs the United Kingdom: P-value 0.010 (moderate significance).

- Italy vs Belgium: P-value 0.015 (moderate significance).

- Italy vs the Netherlands: P-value 0.002 (very strong significance).

* Factor: Farming success and food security

- All countries: P-value < 0.001 (very strong significance).

- Italy vs the United Kingdom: P-value 0.021 (moderate significance).

- Italy vs Belgium: P-value 0.001 (very strong significance).

- France vs Italy: P-value 0.000 (very strong significance).

- France vs The Netherlands: P-value 0.000 (very strong significance).

- France vs Denmark: P-value 0.002 (very strong significance).

- France vs Germany: P-value 0.009 (very strong significance).

- France vs Finland: P-value 0.011 (moderate significance).

- Romania vs The Netherlands: P-value 0.000 (very strong significance).

- Romania vs Italy: P-value 0.000 (very strong significance).

- Romania vs Denmark: P-value 0.002 (very strong significance).

- Romania vs Germany: P-value 0.008 (very strong significance).

- Romania vs Finland: P-value 0.010 (moderate significance).

Footnotes indicate that P-values were adjusted by the Bonferroni correction. Asterisks denote significance levels: two asterisks for 0.01 ≤ P < 0.05 and three asterisks for P < 0.01.

Navigate back to Table 6..

### Table 7. Long description

The table is organized into four columns: Factor, Groups compared, P-value for all groups, and P-value for groupwise comparisons.

1. Food and nature sustainability factor:

- All countries: P-value is less than 0.001 (very strong significance).

- Italy vs the United Kingdom: P-value 0.001.

- Italy vs the Netherlands: P-value 0.013.

- Italy vs France: P-value 0.019.

- Romania vs Belgium: P-value 0.000.

- Romania vs the United Kingdom: P-value 0.000.

- Romania vs the Netherlands: P-value 0.000.

- Romania vs France: P-value 0.000.

- Romania vs Germany: P-value 0.003.

- Romania vs Finland: P-value 0.000.

2. Profitability and success factor:

- All countries: P-value is less than 0.001 (very strong significance).

- The Netherlands vs Finland: P-value 0.015.

- The Netherlands vs Romania: P-value 0.006.

- Italy vs Romania: P-value 0.004.

- Italy vs Finland: P-value 0.020.

Footnotes indicate that significance values were adjusted by the Bonferroni correction. Two asterisks denote moderate evidence (0.01 to 0.05) and three asterisks denote very strong evidence (less than 0.01).

Navigate back to Table 7..

### Table 8. Long description

The table is organized with Country as the first column, followed by two columns for EFA model 1 (citizens) and three columns for EFA model 2 (supply chain actors).

EFA model 1 includes F 1: Health and well-being and F 2: Farming success and food security.

EFA model 2 includes F 1: Health and well-being, F 2: Food and nature sustainability, and F 3: Profitability and success.

Data by country:

* F I (Finland): Citizens F 1 -0.085, F 2 -0.078; Supply chain F 1 0.308, F 2 -0.194, F 3 0.329.

* D K (Denmark): Citizens F 1 -0.080, F 2 -0.054; Supply chain data is N / A.

* R O (Romania): Citizens F 1 0.045, F 2 0.125; Supply chain F 1 0.045, F 2 0.641, F 3 0.345.

* U K (United Kingdom): Citizens F 1 -0.007, F 2 0.055; Supply chain F 1 -0.116, F 2 -0.462, F 3 0.284.

* D E (Germany): Citizens F 1 0.039, F 2 -0.050; Supply chain F 1 0.213, F 2 -0.504, F 3 -0.450.

* B E (Belgium): Citizens F 1 -0.039, F 2 0.067; Supply chain F 1 -0.291, F 2 -0.223, F 3 -0.026.

* N L (Netherlands): Citizens F 1 -0.030, F 2 -0.077; Supply chain F 1 -0.331, F 2 -0.400, F 3 -0.488.

* F R (France): Citizens F 1 -0.027, F 2 0.160; Supply chain F 1 -0.037, F 2 -0.373, F 3 -0.200.

* I T (Italy): Citizens F 1 0.184, F 2 -0.148; Supply chain F 1 0.063, F 2 0.392, F 3 -0.368.

Navigate back to Table 8..
